# Metabotropic Glutamate Receptor 2/3 (mGluR2/3) Activation Suppresses TRPV1 Sensitization in Mouse, But Not Human, Sensory Neurons

**DOI:** 10.1523/ENEURO.0412-17.2018

**Published:** 2018-04-13

**Authors:** Tayler D. Sheahan, Manouela V. Valtcheva, Lisa A. McIlvried, Melanie Y. Pullen, David A.A. Baranger, Robert W. Gereau

**Affiliations:** 1 Washington University Pain Center and Department of Anesthesiology, Washington University School of Medicine, St. Louis, Missouri 63110; 2 Washington University Program in Neuroscience, Washington University School of Medicine, St. Louis, Missouri 63110; 3 BRAIN Laboratory, Department of Psychological and Brain Sciences, Washington University in St. Louis, St. Louis, Missouri 63130

**Keywords:** Dorsal root ganglia, glutamate, human neurons, metabotropic, nociceptors, pain

## Abstract

The use of human tissue to validate putative analgesic targets identified in rodents is a promising strategy for improving the historically poor translational record of preclinical pain research. We recently demonstrated that in mouse and human sensory neurons, agonists for metabotropic glutamate receptors 2 and 3 (mGluR2/3) reduce membrane hyperexcitability produced by the inflammatory mediator prostaglandin E_2_ (PGE_2_). Previous rodent studies indicate that mGluR2/3 can also reduce peripheral sensitization by suppressing inflammation-induced sensitization of TRPV1. Whether this observation similarly translates to human sensory neurons has not yet been tested. We found that activation of mGluR2/3 with the agonist APDC suppressed PGE_2_-induced sensitization of TRPV1 in mouse, but not human, sensory neurons. We also evaluated sensory neuron expression of the gene transcripts for mGluR2 (*Grm2*), mGluR3 (*Grm3*), and TRPV1 (*Trpv1*). The majority of *Trpv1* ^+^ mouse and human sensory neurons expressed *Grm2* and/or *Grm3*, and in both mice and humans, *Grm2* was expressed in a greater percentage of sensory neurons than *Grm3*. Although we demonstrated a functional difference in the modulation of TRPV1 sensitization by mGluR2/3 activation between mouse and human, there were no species differences in the gene transcript colocalization of mGluR2 or mGluR3 with TRPV1 that might explain this functional difference. Taken together with our previous work, these results suggest that mGluR2/3 activation suppresses only some aspects of human sensory neuron sensitization caused by PGE_2_. These differences have implications for potential healthy human voluntary studies or clinical trials evaluating the analgesic efficacy of mGluR2/3 agonists or positive allosteric modulators.

## Significance Statement

Species differences between rodents and humans have been proposed to contribute to the low success rate of analgesic drug development. This work utilizes primary human neurons to assess the translational potential of metabotropic glutamate receptor 2/3 (mGluR2/3), which have been identified as modulators of pain in a variety of rodent models. In mouse sensory neurons, we found that activation of mGluR2/3 blocked inflammation-induced sensitization of the nonselective cation channel TRPV1. In contrast, this effect was not observed in human sensory neurons. These results indicate that mechanisms of peripheral analgesia are not entirely conserved across species. More broadly, our findings demonstrate that using human tissue to validate analgesic targets identified in rodents is an important step in the translational research process.

## Introduction

Species differences between rodents and humans have been proposed to contribute to the low success rate of analgesic drug development (Le Bars et al., 2001; Whiteside and Kennedy, 2010; Woolf, 2010; Mao, 2012). In preclinical research, putative analgesics are often identified and exclusively evaluated in rodent tissues and/or pain models before entering clinical trials, in which such drugs seldom demonstrate efficacy (Kissin, 2010; Woolf, 2010; Moore et al., 2013). Therefore, using primary human neurons to validate preclinical rodent findings is an appealing strategy to improve the translational success of basic pain research findings. With this goal in mind, we and others have established approaches to obtain and use human sensory neurons to better understand human nociceptor physiology (Baumann et al., 1996; Anand et al., 2006; Davidson et al., 2014; Han et al., 2015; Zhang et al., 2015; Sapio et al., 2016; Valtcheva et al., 2016; Rostock et al., 2017).

The group II metabotropic glutamate receptors (mGluRs) have recently been identified as putative targets for pain relief in rodents (Sharpe et al., 2002; Simmons et al., 2002; Yang and Gereau, 2002, 2003; Jones et al., 2005; Du et al., 2008; Osikowicz et al., 2008; Carlton et al., 2009, 2011; Zammataro et al., 2011; Asseri et al., 2015; Kolber, 2015; Chiechio, 2016; Johnson et al., 2017). mGluR2 and mGluR3 are seven transmembrane domain G_i_-protein coupled receptors that decrease cAMP formation, activate potassium channels, and inhibit voltage-gated calcium channels to reduce neuronal excitability and synaptic transmission (Conn and Pin, 1997; Johnson and Schoepp, 2008). Although group II mGluRs are expressed at each level of the pain neuraxis (Petralia et al., 1996; Tang and Sim, 1999; Carlton et al., 2001; Varney and Gereau, 2002; Carlton and Hargett, 2007; Boye Larsen et al., 2014; Kolber, 2015; Chiechio, 2016; Davidson et al., 2016), several lines of evidence suggest that activation of mGlu2 and mGlu3 receptors in peripheral sensory neurons is sufficient for analgesia. For instance, in rodent inflammatory pain models, pharmacological activation of mGluR2/3 expressed on peripheral primary afferents can attenuate pain-like behavior by suppressing sensory neuron sensitization in response to algogens and inflammatory mediators (Yang and Gereau, 2002; Du et al., 2008; Carlton et al., 2011, 2009; Asseri et al., 2015; Davidson et al., 2016). Conversely, pharmacological inhibition of peripheral mGluR2/3 can prolong pain-like behavior and increase sensory neuron activity, suggesting that endogenous activation of mGluR2/3 is analgesic (Yang and Gereau, 2003; Carlton et al., 2011). Given the centrally mediated adverse effects of existing analgesics such as opioid addiction and abuse, peripheral analgesic targets are of particular interest.

Our recent studies on cultured human dorsal root ganglia (DRG) neurons suggest peripheral mGlu2/3 receptors may be clinically relevant analgesic targets. We demonstrated both anatomical and functional expression of group II mGluRs in human DRG (Davidson et al., 2016). Importantly, as in mice, mGluR2/3 activation blocked human nociceptor membrane hyperexcitability produced by the inflammatory mediator prostaglandin E2 (PGE_2_), indicating that a mechanism for peripheral analgesia may be conserved across species (Davidson et al., 2016). Rodent studies suggest that mGlu2/3 receptors expressed on sensory neuron peripheral terminals can also reduce sensory neuron sensitization by suppressing sensitization of TRPV1 (Yang and Gereau, 2002; Du et al., 2008; Carlton et al., 2009, 2011), a nonselective cation channel that detects noxious stimuli and is critical for inflammation-induced peripheral sensitization (Caterina et al., 1997, 2000; Davis et al., 2000; Moriyama et al., 2005). The present study tested whether the same mechanism is conserved in humans. We used sensory neurons obtained from organ donors without chronic pain to determine whether mGluR2/3 activation blocks inflammation-induced sensitization of TRPV1 in human neurons. We demonstrate that group II mGluR activation suppresses PGE_2_-induced sensitization of TRPV1 calcium responses in mouse, but not human, sensory neurons. Interestingly, this functional difference was not explained by species differences in coexpression of the TRPV1 gene transcript with mGlu2 or mGlu3 receptor gene transcripts.

## Materials and Methods

### Animals

All experiments were performed in compliance with protocols approved by the Animal Studies Committee of Washington University in St. Louis (Protocol nos. 20150246 and 20160097). Experiments were conducted on 5–8-wk-old C57BL/6J male and female mice (Jackson Laboratory, RRID:IMSR_JAX000664). Mice were housed in an animal facility with a 12-h light-dark cycle and given food and water *ad libitum*.

### Donors

Human tissue was obtained in compliance with procedures approved by Mid-America Transplant (St. Louis, MO), and the Human Research Protection Office at Washington University in St. Louis provided an International Review Board waiver. Human DRG were obtained from organ donors with full legal consent for use of tissue for research. Only donors without a history of chronic pain were used in this study (Table 1).

**Table 1.** Donor information and tissue uses

Donor	Age, y	Sex	Race	Cause of death	Tissue uses
1	21	Male	White	Stroke	Ca^2+^ imaging
2	55	Female	White	Stroke	Ca^2+^ imaging, FISH
3	22	Male	Black	Anoxia	Ca^2+^ imaging
4	44	Female	White	Stroke	Ca^2+^ imaging
5	12	Female	White	Anoxia	Ca^2+^ imaging, FISH
6	26	Male	White	Head trauma	Ca^2+^ imaging, FISH
7	18	Female	White	Head trauma	Ca^2+^ imaging, FISH
8	18	Male	White	Head trauma	Ca^2+^ imaging, FISH

y, year; FISH, fluorescent in *situ* hybridization.

### Mouse DRG cultures

For each tissue preparation, two age- and sex-matched mice were killed by live decapitation, and cervical through lumbar DRG were removed and pooled together. DRG were incubated in papain (45 U, Worthington) for 20 min at 37°C, 5% CO_2_. DRG were then rinsed and incubated in collagenase (1.5 mg/mL, Sigma-Aldrich) for 20 min. Both enzyme solutions were made up in Ca^2+^- and Mg^2+^-free Hanks’ buffered salt solution (Corning) with 10 mm Hepes (Sigma-Aldrich). DRG were manually triturated with fire-polished Pasteur pipettes (VWR) to dissociate neurons, passed through a 40-μm filter (VWR), and plated onto poly-d-lysine/collagen (Sigma-Aldrich)-coated 12-mm glass coverslips (Thermo Fisher Scientific). Neurons were maintained in culture for 2 d in Neurobasal A medium (Invitrogen) supplemented with 100 U/mL penicillin/streptomycin (Corning), 2 mm GlutaMAX (Life Technologies), 2% B27 (Gibco), and 5% fetal bovine serum (Gibco).

### Human DRG cultures

Human DRG from the first through fifth lumbar vertebrae were surgically extracted and cultured as described in detail previously (Valtcheva et al., 2016). Briefly, after extraction, fat and dura were trimmed away from the ganglia. DRG were minced, incubated in papain for 1 h, rinsed, and incubated in collagenase for 1 h. Both enzyme solutions were made up in an *N*-methyl-d-glucamine artificial cerebrospinal fluid solution (Sigma-Aldrich). DRG were manually triturated with fire-polished Pasteur pipettes to dissociate neurons, passed through a 100-μm filter (VWR), and plated onto poly-d-lysine/collagen–coated 12-mm glass coverslips. Neurons were maintained in culture for up to 9 d in the medium described above (mean time in culture: 6 d). Every 3 d, half of the culture medium was replaced with fresh media to ensure neuronal health.

### Calcium imaging

Calcium imaging experiments were performed on mouse and human sensory neurons on days *in vitro* (DIV) 2 and 3–9, respectively. Acutely after culturing human DRG neurons, satellite glial encased neurons, and thus accurate physiology experiments could not be performed until the glial peeled off and exposed the neuron plasma membrane, which occurred after 3–4 DIV as reported previously (Valtcheva et al., 2016). Mouse calcium imaging experiments were therefore initially performed on DIV 4. Strikingly, we found that only 2% of mouse DRG neurons responded to 100 nm capsaicin on DIV 4, which we interpreted as a functional downregulation of TRPV1. Therefore, we chose to perform mouse calcium imaging experiments on DIV 2 such that recordings were not performed acutely after culturing neurons, yet were completed before TRPV1 functional downregulation.

Cultured neurons from mice and humans were incubated with 3 μg/mL of the ratiometric calcium indicator Fura-2 AM (Life Technologies) for 45 min. Neurons were then incubated in external solution for 15 min to allow for de-esterification of Fura-2 AM. External solution consisted of (in mm): 130 NaCl, 5 KCl, 2 CaCl_2_, 1 MgCl_2_, 30 glucose, and 10 Hepes. For recordings, coverslips were placed into a chamber and perfused with room temperature external solution. Cells were viewed under an inverted microscope (Olympus Optical), and fluorescent images were acquired every 2 s using a Hamamatsu ORCA camera (Hamamatsu). SimplePCI Software (HCImage, Hamamatsu) was used to identify regions of interest surrounding Fura-2 AM–loaded neurons *a priori* and to record fluorescence emission at alternating excitation wavelengths of 357 and 380 nm.

The experimental protocol entailed a 2-min baseline in external solution followed by a 20-s bath application of 100 nm capsaicin (Sigma-Aldrich), a 3-min wash with external solution, then a treatment condition entailing application of either 7 min of vehicle (external solution), 6 min of 1 μm prostaglandin E2 (PGE_2_, Tocris), or 1 min of 10 μm (2R, 4R)-4-aminopyrrolidine-2,4-dicarboxylate (APDC, Tocris) alone followed by 6 min of 10 μm APDC plus 1 μm PGE_2_. Immediately after treatment, a second pulse of 100 nm capsaicin was bath-applied, neurons were washed for 6 min with external solution, and a 10-s pulse of 50 mm KCl was applied to test for cell viability. At least 2 treatment conditions were tested for a given mouse or donor tissue preparation. All drugs were diluted in external solution and bath-applied at a rate of 2 mL/min. Stock solutions of 2.8 mm PGE_2_ and 10 mm APDC were made up in DMSO and water, respectively. Peak calcium responses were calculated by dividing the absolute increase in Fura-2 AM signal after stimulus application by the proceeding 30-s baseline Fura-2 AM signal. The response threshold to capsaicin was defined as an increase of ≥10% from baseline signal. Cells that did not respond to high KCl were excluded from calcium imaging analysis.

### Fluorescent in situ hybridization (RNAscope)

At the conclusion of mouse and human calcium imaging experiments, neurons were fixed on ice with 4% paraformaldehyde/4% sucrose for 15 min and washed with PBS. Fluorescent *in situ* hybridization (FISH) studies were performed according to the protocol for cultured adherent cells using the RNAscope Multiplex Fluorescent Assay (Advanced Cell Diagnostics) with minor modifications. After dehydration and rehydration of cells in ethanol, glass coverslips were mounted onto glass slides using ethyl cyanoacrylate. Neurons were treated with protease III diluted 1:10 (mouse) or 1:5–7.5 (human) at room temperature for 10 min. Species-specific target probes for *Trpv1*, *Grm2*, and *Grm3* (Table 2) were combined, applied to neurons, and allowed to hybridize for 2 h at 40°C in a humidified oven. A series of incubations were then performed to amplify hybridized probe signal and label target probes with the assigned fluorescence detection channel (C1–C3). Coverslips were counterstained with DAPI using ProLong Gold Antifade Mountant (Invitrogen). Neurons were imaged at 40× using a Leica SPE confocal microscope (Leica Microsystems). Fields of interest were identified in the DAPI channel. Fiji (Image J, NIH, RRID: SCR:002285) software was used to calculate neuron diameter and manually quantify single RNA molecule signals. In the RNAscope assay, each punctate dot represents a single target RNA molecule. However, to reduce the likelihood of false positives, mouse and human neurons were defined as positive for a given RNA target if they had ≥4 or ≥2 puncta, respectively, based on the range in neuron puncta density observed for each species. Although we used the same mouse and human tissues used in both assays (Table 1), neuron populations analyzed using FISH were not identical to those analyzed via calcium imaging.

**Table 2.** RNAscope probes used for FISH

Target	Catalog no.
Mm-Trpv1	313331
Mm-Grm2-C3	317831-C3
Mm-Grm3-C2	317821-C2
Positive Control Probe-Mm	320881
Hs-TRPV1	415381
Hs-GRM2-C3	589771-C3
Hs-GRM3-C2	500181-C2
Positive Control Probe-Hs	320861
Negative Control Probe	320871

Mm, mus musculus; Hs, homo sapiens; C2, channel 2; C3, channel 3.

### Statistical analyses

The experimenter was blind to treatment condition and gene of interest throughout analysis of calcium imaging and *in situ* hybridization data, respectively. Microsoft Excel, GraphPad Prism, R, and the R package Ime4 (RRID:SCR_015654) were used for data organization and statistical analyses (Bates et al., 2014; Team, 2017). Calcium imaging data were analyzed using (1) unpaired *t* tests and Bonferroni correction for multiple comparisons and (2) linear mixed-effects model (LMM) analyses. LMM analyses controlled for donor as a random effect and donor sex, donor age, and DIV as fixed effects. As the primary objective of the present study was to make species comparisons, we were not sufficiently powered to evaluate the effects of mouse age or sex on calcium imaging outcomes. Species comparisons of the percentage of capsaicin-responsive neurons and gene transcript expression were made using χ^2^ tests and Bonferroni correction for multiple comparisons, when appropriate. When describing and discussing species differences in gene transcript expression, we default to mouse mRNA nomenclature. Superscript letters listed with *p*-values correspond to the statistical tests shown in Table 3.

**Table 3.** Statistical analysis

Location	Data Structure	Type of Test	Comparison	95% confidence interval
a	Non-normally distributed	**t**test	Vehicle vs. PGE_2_	–0.6862 to –0.2728
b	Non-normally distributed	**t**test	PGE_2_ vs. PGE_2_ + APDC	0.1317 to 0.618
	Non-normally distributed	**t**test	Vehicle vs. PGE_2_ + APDC	–0.2736 to 0.06428
c	Non-normally distributed	**t**test	Vehicle vs. PGE_2_	–0.5774 to –0.2563
d	Non-normally distributed	**t**test	PGE_2_ vs. PGE_2_ + APDC	–0.4204 to 0.2717
	Non-normally distributed		Vehicle vs. PGE_2_ + APDC	–0.7953 to –0.1871
e	Non-normally distributed	Regression	Vehicle vs. PGE_2_ vs. PGE_2_ + APDC	–0.1178 to 0.1284
f	Categorical	Chi-squared^a^		0.5621 to 0.8235
g	Mouse: non-normally distributed	**t**test	Mouse vs. human	–15.17 to –13.08
	Human: normally distributed			
h	Categorical	Chi-squared^a^		2.653 to 4.769
i	Categorical	Chi-squared^a^		1.846 to 3.855
j	Categorical	Chi-squared^a^		1.047 to 1.967

The D’Agnostino and Pearson normality test was performed, when applicable.

^a^Odds ratio confidence interval reported.

## Results

### mGluR2/3 suppress PGE_2_-induced TRPV1 sensitization in mouse, but not human, sensory neurons

**Figure 1.**
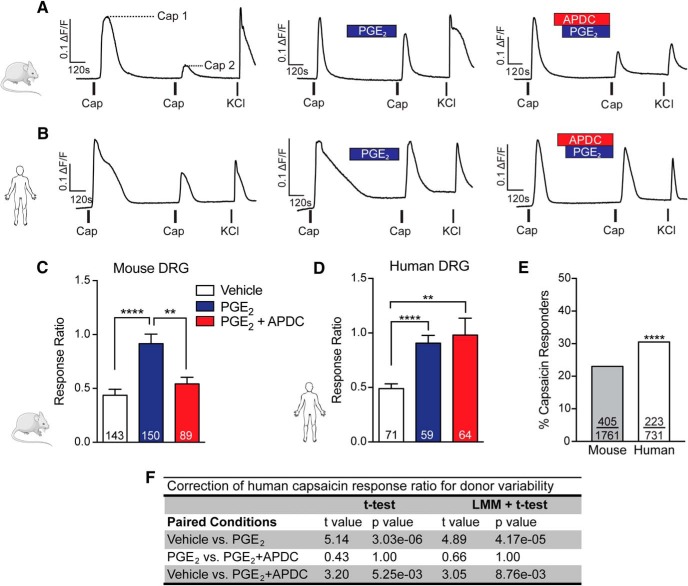
mGlu2/3 receptor activation blocks PGE_2_-induced TRPV1 sensitization in mouse, but not human, sensory neurons. Representative traces of 100 nm capsaicin (Cap)-induced calcium responses in mouse (***A***) and human (***B***) DRG neurons in response to vehicle (left), 1 μm PGE_2_ (middle), or 1 μm PGE_2_ + 10 μm APDC (right). Experiments concluded with a pulse of 50 mm KCl to determine cell viability. The degree of TRPV1 sensitization is expressed as a response ratio calculated by dividing the peak amplitude of Cap 2 by the peak amplitude of Cap 1 (***A***, dashed lines). ***C***, In mouse DRG neurons, PGE_2_ significantly increased the capsaicin response ratio compared with vehicle (**** *p* = 2.1 × 10^–5^; *n* = 143–150 neurons, *n* = 4 preps/condition). Coapplication of APDC with PGE_2_ blocked this effect and significantly reduced the response ratio compared to PGE_2_ alone (** *p* = 0.0081; *n* = 89–150 neurons, *n* = 3–4 preps/condition). ***D***, PGE_2_ also significantly increased the capsaicin response ratio of human DRG neurons compared with vehicle (**** *p* = 3.0 × 10^–6^; *n* = 59–71 neurons, *n* = 5–6 donors/condition); whereas coapplication of APDC did not suppress PGE_2_-induced increases in the capsaicin response ratio (*p* = 1, *n* = 59–64 neurons, *n* = 6 donors/condition), which remained significantly greater than vehicle (** *p* = 0.0053, *n* = 64–71 neurons, *n* = 5–6 donors/condition). Capsaicin response ratios were compared using unpaired *t* tests and a Bonferroni correction for multiple comparisons. Data are presented as mean ± SEM. ***E***, Compared with those of mice, a greater percentage of human DRG neurons responded to 100 nm capsaicin (χ^2^ = 15.45, **** *p* = 8.5 × 10^–5^, mouse: 405/1761 neurons, *n* = 4 preps, human: 223/731 neurons, *n* = 8 donors). ***F***, LMM regression correction for impact of individual donor, as well as donor age and sex, did not alter human capsaicin response ratio analysis statistical outcomes compared with *t* tests alone.

TRPV1 is sensitized by the cAMP/PKA pathway, which is stimulated by inflammatory mediators such as PGE_2_ (Lopshire and Nicol, 1998; De Petrocellis et al., 2001; Bhave et al., 2002; Mohapatra and Nau, 2003; Meves, 2006). In contrast, group II mGlu receptor activation inhibits adenylyl cyclase and subsequent cAMP production (Conn and Pin, 1997; Johnson and Schoepp, 2008). To determine whether mGluR2/3 activation blocks TRPV1 sensitization, we quantified capsaicin-induced calcium responses of mouse and human sensory neurons. Two 20-s pulses of 100 nm capsaicin were bath-applied to DRG neurons, and the degree of TRPV1 sensitization was defined as the response ratio of the peak of the second capsaicin response divided by the peak of the first capsaicin response. Under vehicle conditions, TRPV1 desensitization is observed in both mouse and human DRG neurons in the form of reduced calcium responses to subsequent capsaicin pulses (Fig. 1*A*, *B*). In mouse sensory neurons, bath application of PGE_2_ between capsaicin pulses significantly increased the capsaicin response ratio compared with vehicle (Fig. 1*A*, *C* ^a^). Coapplication of the selective group II mGluR agonist APDC with PGE_2_ significantly reduced the response ratio compared with PGE_2_ alone (Fig. 1*A*, *C* ^b^). As an additional measure of TRPV1 sensitization, we quantified the total calcium load, or area under the curve (AUC) of a subset of capsaicin responses. Consistent with the effects on capsaicin peak response ratios, application of PGE_2_ significantly increased AUC compared with vehicle, and coapplication of APDC with PGE_2_ significantly reduced this effect (data not shown). These findings confirm our previously published work in cultured sensory neurons obtained from CD-1 mice in which we also demonstrated that suppression of PGE_2_-induced TRPV1 sensitization by APDC is blocked by the group II mGluR antagonist LY341495, and thus is attributable to mGlu2/3 receptor activation (Yang and Gereau, 2002).

In human sensory neurons, we found that although application of PGE_2_ between capsaicin pulses significantly increased the capsaicin response ratio compared with vehicle, the response ratio after coapplication of APDC with PGE_2_ did not significantly differ from application of PGE_2_ alone (Fig. 1*B*, *D* ^c,d^). We observed similar results for total calcium load of capsaicin responses (data not shown). Unlike rodents used in preclinical studies, human organ donors display wide demographic and genetic diversity. To correct for between-donor heterogeneity, we performed a LMM regression. As the effect of age and sex on human sensory neuron physiology is of broad interest to pain researchers, these parameters were included as covariates in our LMM regression. Interestingly, we found that correcting for impact of individual donor, as well as donor age and sex, did not alter our capsaicin response ratio outcome (Fig. 1*F*). Moreover, LMM regression analysis indicated that the day *in vitro* at which calcium imaging experiments were performed did not influence capsaicin response ratios for any of the conditions tested (*t* = 0.105, *p* = 0.9182^e^). Taken together, these results demonstrate that mGluR2/3 activation suppresses PGE_2_-induced TRPV1 sensitization in mouse, but not human, sensory neurons.

We were also interested in whether the same percentage of mouse and human sensory neurons respond to capsaicin. A neuron was defined as capsaicin responsive if it responded to either the first or second pulse of capsaicin. Notably, compared with vehicle conditions, application of PGE_2_ did not increase the number of neurons that did not respond to the first pulse of capsaicin but did respond to the second pulse of capsaicin in either species. Therefore, data were pooled across treatment conditions for calculations of percentage of capsaicin-responsive neurons. Of the total number of sensory neurons evaluated in calcium imaging studies, 23.0% of mouse sensory neurons versus 30.5% of human sensory neurons responded to 100 nm capsaicin, indicating that human sensory neurons are modestly more capsaicin responsive (Fig. 1*E* ^f^). These results are consistent with our initial observations that human sensory neurons exhibit greater chemosensitivity to algogens and pruritogens compared with rodents (Davidson et al., 2014). In the present study, we chose to use 100 nm capsaicin to test for modulation of TRPV1 desensitization. Previous studies using higher concentrations of capsaicin (200 nm to 1 µm) indicate that capsaicin elicits calcium responses in a greater proportion of mouse DRG and trigeminal neurons, ranging from 30% to 70% (Davis et al., 2000; Elitt et al., 2008; Barabas and Stucky, 2013; Ren et al., 2014; Valtcheva et al., 2015; Mohammed et al., 2017).

### *Mouse and human sensory neurons share similar* Trpv1, Grm2*, and* Grm3 *expression and coexpression profiles*

**Figure 2.**
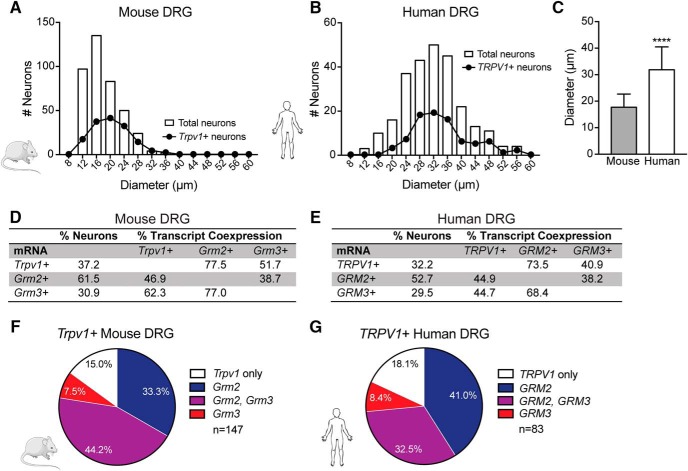
Expression of *Trpv1*, *Grm2*, and *Grm3* mRNA transcripts in dissociated mouse and human sensory neurons. ***A***, Size distribution of total and *Trpv1* ^+^ mouse DRG neuron populations; mean diameter of total neurons: 17.7 ± 0.2 μm (*n* = 395 neurons, *n* = 4 preps), mean diameter of Trpv1^+^ neurons: 20.3 ± 0.4 μm (*n* = 147 neurons). ***B***, Size distribution of total and TRPV1^+^ human DRG neuron populations; mean diameter of total neurons: 31.9 ± 0.5 μm (*n* = 258 neurons, *n* = 5 donors), mean diameter of TRPV1^+^ neurons: 33.9 ± 0.9 μm (*n* = 83 neurons). Data are reported as mean ± SEM. ***C***, The mean diameter of total human DRG neurons was significantly larger than that of total mouse DRG neurons (unpaired *t* test, **** *p* = 1.0 × 10^–14^). Data are presented as mean ± SD. Percentage of total mouse (***D***) and human (***E***) DRG neurons that expressed *Trpv1*, *Grm2*, and *Grm3*, as well as the percentage of neurons that coexpressed one mRNA transcript with another. Pie charts showing the percentage of *Trpv1* ^+^ mouse (***F***) and human (***G***) DRG neurons that coexpressed *Grm2*, *Grm3*, or both transcripts. No significant differences in gene transcript expression of total neurons or *Trpv1* ^+^ neuron subpopulations were observed between species.

We hypothesized that the observed species differences in mGluR2/3 functional modulation of TRPV1 could be due to reduced coexpression between TRPV1 and mGlu2 and/or mGlu3 receptors in human versus mouse sensory neurons. We previously demonstrated mGluR2 immunoreactivity in human sensory neurons (Davidson et al., 2016). However, because of the lack of highly selective mGluR3 antibodies suitable for immunohistochemistry (García-Bea et al., 2016), we assessed expression of TRPV1, mGluR2, and mGluR3 mRNA transcripts (referred to as *Trpv1*, *Grm2*, and *Grm3*, respectively) in dissociated sensory neurons using RNAscope FISH. The mouse DRG neurons evaluated in FISH studies ranged from 10.0 to 35.5 μm in diameter, with a mean diameter of 17.7 ± 0.2 μm for the total population and 20.3 ± 0.4 μm for *Trpv1* ^+^ neurons (Fig. 2*A*). In comparison, human DRG neurons ranged from 10.0 to 56.3 μm in diameter, with a mean diameter of 31.9 ± 0.5 μm for the total population and 33.9 ± 0.9 μm for *TRPV1* ^+^ neurons (Fig. 2*B*). The mean diameter of the total human DRG neuron population was significantly larger than that of mice (Fig. 2*C* ^g^). Our findings closely resemble the size distribution of mouse DRG neurons reported previously (Dirajlal et al., 2003; Barabas et al., 2012; O’Brien et al., 2015). Previous human studies show that the average sensory neuron diameter in unfixed tissues is between ∼40 and 60 μm (Anand et al., 2006; Davidson et al., 2014; Xu et al., 2015; Han et al., 2016; Zhang et al., 2017), further highlighting the species difference in sensory neuron size.

With respect to mRNA expression, of the total population of mouse DRG neurons, 37.2% of neurons were positive for the *Trpv1* gene transcript, 61.5% were positive for *Grm2*, and 30.9% were positive for *Grm3* (Fig. 2*D*). Similar transcript expression was observed in human sensory neurons, with 32.2% of all neurons positive for *TRPV1*, 52.7% positive for *GRM2*, and 29.5% positive for *GRM3* (Fig. 2*E*). Notably, a significantly greater percentage of total DRG neurons expressed *Grm2* compared to *Grm3* in both mice (χ^2^ = 74.56, *p* = 1.0 × 10^–15 h^) and humans (χ^2^ = 28.83, *p* = 8.0 × 10^–8 i^). Although there was a trend toward an increased percentage of *Grm2* ^+^ neurons in mouse compared with human DRG (χ^2^ = 2.229 with Bonferroni correction, *p* = 0.077^j^), there were no significant species differences in the expression of the transcripts of interest.

Analysis of transcript coexpression showed that the majority of *Trpv1* ^+^ mouse DRG neurons coexpressed *Grm2*, *Grm3*, or both. To elaborate, 33.3% of *Trpv1* ^+^ neurons coexpressed *Grm2*, 7.5% coexpressed *Grm3*, and 44.2% coexpressed both *Grm2* and *Grm3* (Fig. 2*F*). Of *TRPV1* ^+^ human DRG neurons, 41.0% coexpressed *GRM2*, 8.4% coexpressed *GRM3*, and 32.5% coexpressed both *GRM2* and *GRM3* (Fig. 2*G*). No significant species differences were observed in the coexpression of *Trpv1* with *Grm2* and/or *Grm3* gene transcripts. Thus, these findings suggest that the absence of mGluR2/3-induced suppression of TRPV1 sensitization in human DRG neurons cannot be explained by reduced coexpression of TRPV1 with group II mGluRs at the gene transcript level.

## Discussion

Peripheral targets for pain relief are greatly desired given the centrally mediated side effects, including addiction and misuse of current frontline analgesics such as opioids. In both mice and humans, activation of group II mGluRs blocks sensory neuron membrane hyperexcitability elicited by the inflammatory mediator PGE_2_ (Davidson et al., 2016). In the present study, we demonstrate that suppression of inflammation-induced TRPV1 sensitization represents an additional mechanism by which mGluR2/3 reduce peripheral sensitization in mouse, but not human, sensory neurons. We further show equivalent colocalization of *Trpv1* with *Grm2* and *Grm3* mRNA transcripts in mouse and human DRG neurons, suggesting that disparities in coexpression do not explain species differences in the functional modulation of TRPV1 by group II mGluRs. These findings indicate that although mGluR2/3 activation decreases sensory neuron sensitization in both mice and humans, mechanisms of peripheral analgesia are not fully conserved across species.

### mGluR2/3 functional differences in mouse and human sensory neurons

That the mGlu2/3 receptor agonist APDC did not suppress PGE_2_-induced TRPV1 sensitization in human DRG neurons was a surprising observation. Foremost, cDNA and amino acid sequences of human and rodent mGluR2 and mGluR3 display at least 90% homology (Flor et al., 1995; Makoff et al., 1996; Johnson and Schoepp, 2008). In turn, APDC exhibits comparable potency at rodent and human group II mGluRs with respect to inhibition of stimulated cAMP responses (Schoepp et al., 1995). Our recent finding that APDC decreases excitability and increases action potential threshold in PGE_2_-treated sensory neurons of both species further suggests mGluR2/3 functional homology in mouse and human sensory neurons (Davidson et al., 2016). Existing behavioral and *in vitro* rodent studies strongly suggest that inhibition of cAMP-dependent TRPV1 sensitization is another mechanism by which mGluR2/3 can block sensory neuron sensitization (Yang and Gereau, 2002; Du et al., 2008; Carlton et al., 2009, 2011). Here, we substantiate these findings by demonstrating that APDC blocks PGE_2_-induced TRPV1 sensitization in C57BL/6J mice. However, this observation did not translate in human sensory neurons despite the apparent similarities of mGluR2/3 function between species.

There are multiple potential explanations for the lack of translation of mGluR2/3 functional modulation of TRPV1 from mice to humans. For example, in addition to PKA, PGE_2_-induced intracellular signaling cascades can activate other kinases known to sensitize TRPV1, including PKC and c-Src kinase (Vellani et al., 2001; Numazaki et al., 2002; Jin et al., 2004; Moriyama et al., 2005). Thus, although we observed PGE_2_-induced sensitization of capsaicin responses in both mouse and human sensory neurons, it is possible that in contrast to mice, PGE_2_-induced TRPV1 sensitization in humans occurs via a predominantly PKA-independent pathway that is not influenced by mGlu2/3 receptor activation. Further investigation of the intracellular mechanisms that underlie PGE_2_-induced TRPV1 sensitization in human sensory neurons is therefore needed.

Another important consideration is that although we observed equivalent coexpression of *Grm2* and *Grm3* gene transcripts in *Trpv1^+^* mouse and human DRG neurons, whether equivalent coexpression of TRPV1 and mGluR2/3 also extends to the protein level remains unclear. For instance, it is possible that species differences exist in the regulation of translation, posttranslational modifications, and subcellular compartmentalization of TRPV1 and mGlu2/3 receptors. TRPV1 immunoreactivity has been demonstrated in human DRG neurons, peripheral nerves, and intra-epidermal nerve fibers (Anand et al., 2006, 2015; Facer et al., 2007; Li et al., 2015; Han et al., 2016). However, until selective mGluR2 and mGluR3 antibodies suitable for immunohistochemistry are generated, our ability to evaluate the coexpression and subcellular localization of group II mGluRs with TRPV1 remains limited. Importantly, although mGlu2/3 receptor activation does not modulate sensitization of TRPV1 in humans, the expression of *GRM2* and/or *GRM3* in the majority of small-diameter (<50 μm) *TRPV1* ^+^ human DRG neurons suggests that these receptors are well positioned to modulate nociceptor activity by alternative mechanisms. Thus, mGluR2/3 remain putative human peripheral analgesic targets.

### Sensory neuron expression of Grm2, Grm3, and Trpv1

Existing immunohistochemical analyses of rodent DRG neurons demonstrate high colocalization of group II mGlu receptors with TRPV1, with 93% of TRPV1-positive neurons expressing mGluR2/3 and effectively all mGluR2/3-positive neurons expressing TRPV1 (Carlton et al., 2009). Here we demonstrate that the majority (≥81%) of *Trpv1* ^+^ mouse and human sensory neurons also express *Grm2* and *Grm3* gene transcripts. In contrast, we found that in both mice and humans, only a subset (45%–62%) of either *Grm2* ^+^ or *Grm3* ^+^ neurons also expressed the *Trpv1* transcript. Importantly, prior immunohistochemistry studies using nonselective mGluR2/3 antibodies and RNA-sequencing analysis of homogenized DRG precluded analyses of which group II mGlu receptor is predominantly expressed in sensory neurons and to what extent mGluR2 and mGluR3 are coexpressed within the same neurons. We show for the first time that *Grm2* is more highly expressed than *Grm3* in mouse and human sensory neurons. Further, while *Grm2* and *Grm3* are coexpressed in a subset (38%–77%) of mouse and human sensory neurons, the transcripts are also expressed individually. These findings suggest that mGluR2 may play a larger role in modulating nociceptor excitability than mGluR3. Indeed, previous behavioral studies demonstrate that the analgesic efficacy of group II mGlu receptor agonists persists in mGluR3^-/-^, but not mGluR2^-/-^, mice, suggesting a greater role for mGluR2 than mGluR3 in pain regulation (Zammataro et al., 2011). As repeated dosing with group II mGlu receptor agonists causes analgesic tolerance in rodents (Jones et al., 2005; Chiechio et al., 2009), alternative strategies to reinforce endogenous activation of mGluR2 may be required to effectively target group II mGlu receptors for clinical pain relief. Our results suggest that recently developed mGluR2-specific positive allosteric modulators may be promising agents for blockade of peripheral sensitization (Galici et al., 2006; Asseri et al., 2015).

### Distinctions between human and rodent sensory neurons

In addition to differences in mGlu2/3 receptor function, we demonstrate that human sensory neurons possess distinct fundamental properties including increased diameter and an increased percentage of capsaicin-responsive neurons compared with those of mice. Using calcium imaging, we found that 30.5% of human DRG neurons responded to 100 nm capsaicin. However, it is possible that a larger proportion of human sensory neurons expresses TRPV1 and/or responds to capsaicin. For instance, previous immunohistochemical analysis of human DRG showed that roughly 55% of all DRG neurons were TRPV1-immunoreactive (Anand et al., 2006). Further, in calcium imaging experiments, ∼60% of ganglionectomized DRG neurons removed due to chronic intractable pain responded to 100 nm capsaicin (Baumann et al., 1996, 2004). These findings suggest that an even greater species difference in capsaicin-responsive neurons may exist between mice and humans than we report here. Of course, variability in experimental conditions or donor demographics, genetic diversity, and pain-related health conditions could underlie differences in TRPV1 expression across human studies. For this reason, developing an extensive donor tissue bank to investigate DRG neuron gene and protein expression as well as to further characterize sensory neuron subpopulations will allow for more complete comparisons between species, and perhaps more interestingly, among donor subpopulations.

Nevertheless, while only a small number of comparative studies of rodent and human sensory neurons have been conducted, it is becoming increasingly clear that species differences exist in gene expression, ion channel properties, and action potential firing patterns (Baldo et al., 2013; Han et al., 2015; Chang et al., 2017; Ray et al., 2017; Rostock et al., 2017; Zhang et al., 2017). Therefore, human sensory neurons represent a vital tool for improving our understanding of human nociceptor physiology under both normal and pathologic conditions. Further, using human sensory neurons to assess the validity of putative analgesic targets identified in rodents may lead to increased translational success of preclinical findings.

## Synthesis

Reviewing Editor: Mary Heinricher, Oregon Health & Science University Decisions are customarily a result of the Reviewing Editor and the peer reviewers coming together and discussing their recommendations until a consensus is reached. When revisions are invited, a fact-based synthesis statement explaining their decision and outlining what is needed to prepare a revision will be listed below. The following reviewer(s) agreed to reveal their identity: Gregory Dussor, Derek Molliver.

The validity of rodent models and their translatability to humans is a critical issue in the development of novel therapeutics. In the pain field, the use of human dorsal root ganglia for in vitro electrophysiology and imaging experiments allows direct tests of mechanisms between other species and humans. The current manuscript performs such a comparative, focusing specifically on modulation of TRPV1 sensitization through activation of mGluR2/3 receptors. Using ratiometric calcium imaging, the authors show that application of an exogenous agonist at mGluR2/3 receptors interferes with sensitization of DRG responses in mouse, but not human, cultured DRG neurons. This was despite an apparent similarity in receptor expression. These findings have direct implications for possible development of mGluR2/3 receptor agonists for pain in humans, but more broadly, highlight the value of physiological studies in human neurons when validating potential therapeutic targets.

Given the implications of these observations for both drug development and the usefulness of mouse models, reviewers agreed that it is critical to ensure that the findings are valid. One important difference between the mouse and human studies was the time in culture. Mouse DRG neurons were used at 2 days in culture, while human neurons were used up to 9 days in culture (average of 6 days). Could this difference in culture time have contributed to the apparent species differences? While it is true that there was no difference in mRNA at the different time points, we don't know that the proteins were expressed and had equivalent function over time. One way of getting at this question would be a demonstration that APDC decreases TRPV1 sensitization in mouse at later time points. (That is, do mouse neurons still show the same responses at days 6 to 9 in vitro as at day 2?)

A related question is whether the differences in the sizes of the neurons analyzed from the two species could play a role in the apparent species difference. The mean diameter of mouse neurons was 17.7 um, while that of human neurons was 31.9 um. Could this have contributed to the difference.

There are some additional issues that would benefit from clarification, additional analysis, or deeper discussion.

The authors report peak calcium transients. Analysis of total calcium load (AUC) or decay time might provide additional sensitivity.

Were the neurons used for mRNA analysis that specific neurons selected for calcium measurements? Or was the entire dish analyzed for mRNA and only a fraction of the cells used for calcium measurements? If the latter, it is possible that changes in the neurons used for calcium imaging were not representative.

The authors note that higher concentrations of capsaicin have yielded higher percentages of responsive neurons in other rodent studies (p11). Was there a subset of human/mouse neurons in the current study that did not initially respond to capsaicin but became responsive after exposure to PGE2?

Was there a significant difference in the magnitude of the responses to KCl in cells that received PGE2 versus those that received PGE2 plus APDC, which might indicate an impact of G protein signaling on voltage-gated sodium or calcium channels? Was this measure different between mouse and human neurons?

Readers might find a reference to Rostock et al., (Neuroscience, 2017) useful. In addition, a discussion of how the findings of this and their prior paper (Davidson, et al., 2016) differ, and how that might impact their conclusions.
